# Endocan, a Risk Factor for Developing Acute Respiratory Distress Syndrome among Severe Pneumonia Patients

**DOI:** 10.1155/2019/2476845

**Published:** 2019-04-01

**Authors:** Jun Ying, Danfei Zhou, Tongjie Gu, Jianda Huang

**Affiliations:** Department of Respiratory, Hwa Mei Hospital, University of Chinese Academy of Sciences, No. 41, Xibei Street, Ningbo 315000, Zhejiang, China

## Abstract

**Background:**

Severe pneumonia (SP) has been widely accepted as a major cause for acute respiratory distress syndrome (ARDS), and the development of ARDS is significantly associated with increased mortality. This study aimed to identify potential predictors for ARDS development in patients with SP.

**Methods:**

Eligible SP patients at admission from January 2013 to June 2017 were prospectively enrolled, and ARDS development within hospital stay was identified. Risk factors for ARDS development in SP patients were analyzed by univariate and multivariate logistic regression analysis. The receiver operating characteristic (ROC) curve analysis with the area under the curve (AUC) was performed for the predictive value of endocan for ARDS development.

**Results:**

A total of 145 SP patients were eventually enrolled into the final analysis, of which 37 developed ARDS during the hospital stay. Our final multivariate logistic regression analysis suggested plasma endocan expression as the only independent risk factor for ARDS development in SP patients (OR: 1.57, 95% CI: 1.14–2.25, *P*=0.021). ROC curve analysis of plasma endocan resulted in an AUC of 0.754, 95% CI of 0.642–0.866, a cutoff value of 11.6 ng/mL, a sensitivity of 78.7%, and a specificity of 70.3%, respectively (*P* < 0.01).

**Conclusions:**

Endocan expression at ICU admission is a reliable predictive factor in predicting ARDS in patients with SP.

## 1. Introduction

Acute respiratory distress syndrome (ARDS), a common devastating problem encountered in critically ill patients, is closely associated with acute respiratory failure, limited life quality, and high mortality [1, 2]. Previous studies have revealed an incidence of ARDS among patients admitted to the intensive care unit (ICU) as high as 10% [3]. Severe pneumonia (SP) has been widely accepted as a major cause for ARDS, and the development of ARDS is significantly associated with increased mortality [4]. The overlap of clinical symptoms in SP and ARDS results in the difficulty of identification of ARDS from SP [5]. Therefore, the early predication and risk stratification of ARDS in patients with SP is of great importance to improve the prognosis. However, the data indicating risk factors for the development of ARDS during the course still remain sparse.

The morphological and functional alteration of pulmonary endothelium is one of the great characteristics of ARDS and closely associated with the high mortality [6, 7]. Endocan, a proteoglycan mainly expressed in pulmonary microcirculation, plays a critical role in endothelial homeostasis by the modulation of leukocyte migration, endothelial permeability, and cell adhesion [8]. As firstly described by Kao et al., a positive correlation has been observed between blood endocan and pneumonia severity index (PSI) [9]. In a cohort of patients with a diagnostic of ARDS, Orbegozo et al. [10] have reported higher endocan levels in patients with poor outcomes, suggesting an association between elevated endocan values and severity of ARDS. However, few data have indicated whether endocan can serve as a predicator for ARDS development in patients with SP. We aimed to identify potential predictors for ARDS development in patients with SP in this present study.

## 2. Materials and Methods

### 2.1. Patients

This study was approved by the Medical Institutional Ethics Committee of Zhejiang Province and Hwa Mei Hospital, University of Chinese Academy of Sciences. Eligible patients admitted to the Department of Respiratory Intensive Care Unit (RICU) of Hwa Mei Hospital, University of Chinese Academy of Sciences, from January 2013 to June 2017 were enrolled. Inclusion criteria were described as follows: (1) with the diagnosis of SP according to the consensus put forward by Infectious Diseases Society of America and American Thoracic Society [11]; (2) adult patients (aged over 18 years); and (3) with written informed consent. Those patients with pregnancy, neuromuscular diseases, alternative pulmonary diseases (including pneumothorax, asthma, acute exacerbation of chronic obstructive pulmonary disease, and pulmonary thromboembolism), and ARDS were excluded. Patients with immunosuppressive therapy or aggressive carcinoma were also excluded.

### 2.2. Treatment Strategies

Blood and sputum cultures on admission were conducted for pathogens identification. Antimicrobial therapies (empirical and subsequent pathogen-directed) were appropriately administered under the directions of the consensus guidelines [12]. Mechanical ventilation (MV), circulation stabilization, nutrition support, organ function support, and internal environment maintenance were implemented according to the corresponding guidelines.

### 2.3. Demographic and Clinical Characteristics

To screen potential risk factors for ARDS development, we collected the demographic, clinical, and diagnostic characteristics on admission. The primary endpoint was the development of ARDS which was defined according to the Berlin definition [13] during the hospital stay. Demographic and clinical characteristics including age, gender, smoking habits, clinical scores, comorbidities, presence of sepsis, body temperature, respiratory rate, heart rate, systolic blood pressure (SBP), diastolic blood pressure (DBP), SpO_2_, presence of pleural effusion, and treatment strategies were recorded in details.

### 2.4. Laboratory Tests

Arterial blood was sampled on admission for the blood gas analysis. Fasting venous blood samples were also obtained on admission for the analyses of blood cell counting, inflammation biomarkers, and T-cell immunity, and biochemical analysis.

### 2.5. Endocan Measurement

Fasting venous blood samples were taken on admission for the measurement. The obtained blood samples in tubes containing with EDTA were immediately centrifuged, and subsequently, the separated plasma samples were frozen at −80°C for further analysis. Plasma endocan expressions were detected by the method of enzyme-linked immunosorbent assay (ELISA) with anti-human endocan antibodies (Lunginnov, Lille, France). The plasma inflammatory cytokines (CRP, IL-6, and procalcitonin) were also measured by ELISA according to the manufacturer's instructions (R&D Systems, CA, USA). The measurement of plasma endocan was carried out by following the guidance of manufacturer's instructions using the human endocan kit (JDIEK kit H1, LIK-1205, Lunginnov, Lille, France). The main characteristics are as follows: detection limit 0.15 ng/ml, the limit of quantification 0.3 ng/ml, intra-assay CV 4.80%, interassay CV 7.59%, and blood value in healthy controls 0.15 to 2.5 ng/mL [14].

### 2.6. Statistical Analysis

The statistical analysis was administered using GraphPad prism 5.0 (GraphPad Inc., San Diego, CA, USA) and SPSS 19.0 (SPSS Inc., Chicago, IL, USA). Continuous variables were presented as mean ± standard deviation (SD), while dichotomous variables with number and proportion. Student's *t*-test, Mann–Whitney *U*-test, chi-squared test, or Fisher's exact test were conducted for data comparison as appropriate. All the potential risk factors by previous analyses were subsequently enrolled into the univariate logistic regression analysis. To identify potential predictors for the development of ARDS, only those risk factors with a *P* value < 0.1 by the univariate logistic analysis were verified by the multivariate logistic regression. The receiver operating characteristic (ROC) curve analysis with the area under the curve (AUC) was performed for the predictive value of endocan for ARDS development. *P* < 0.05 was considered as statistically different.

## 3. Results

A total of 145 SP patients were eventually enrolled into the final analysis, of which 37 developed ARDS during the hospital stay with the average time of 7.1 days. The percentage of SP patients who developed ARDS was 25.5% in this present study, which was quite in accordance with other results [15]. The demographic and clinical characteristics are summarized in Table 1. The sequential organ failure assessment (SOFA) score was calculated to be lower, while the lung injury score (LIS) was higher in those SP patients who developed ARDS. Patients with ARDS development were older and had higher body temperature, heart rate, SBP, and SpO_2_ than those without ARDS development. However, smoking habits, acute physiology and chronic health evaluation (APACHE) II score, comorbidities, presence of sepsis or pleural effusion, respiratory rate, and treatment strategies did not differ significantly in SP patients with or without ARDS development.

As shown in Table 2, plasma endocan expressions were statistically significantly higher in patients with ARDS development than in those without ARDS development. No statistically significant differences were observed in the comparison of blood cell analysis in patients with or without ARDS. Patients with higher concentrations of BUN, creatinine, lactate, and CRP were significantly associated with the development of ARDS.

**Figure 1:**
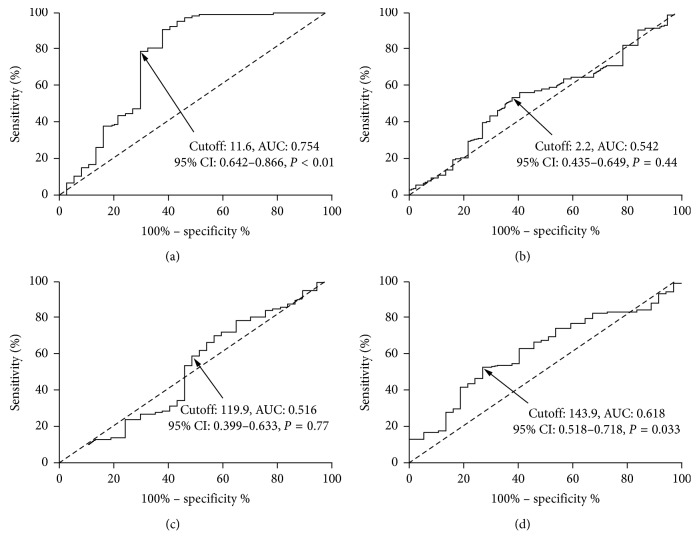
ROC curve analyses of plasma endocan (a), LIS (b), CRP (c), and PaO_2_/FiO_2_ ratio (d) to predict ARDS development. Plasma endocan expression on admission was a potential predictor for ARDS development with an AUC of 0.754, a 95% CI of 0.642–0.866, a cutoff value of 11.6 ng/mL, a sensitivity of 78.7%, and a specificity of 70.3%, respectively (*P* < 0.01). PaO_2_/FiO_2_ ratio was also a potential predictor for ARDS development (cutoff value: 143.9, AUC: 0.618, 95% CI: 0.518–0.718, sensitivity: 53.7%, specificity: 67.6%, and *P*=0.033). ROC, receiver operating characteristic; AUC, area under the curve; CI, confidence interval; LIS, lung injury score; CRP, C-reactive protein; PaO_2_/FiO_2_, ratio of partial pressure of arterial oxygen to fraction of inspired oxygen.

To discriminate ARDS from SP, ROC curve analysis of plasma endocan resulted in an AUC of 0.754, 95% CI of 0.642–0.866, a cutoff value of 11.6 ng/mL, a sensitivity of 78.7%, and a specificity of 70.3%, respectively (shown in Figure 1(a), *P* < 0.01). PaO_2_/FiO_2_ ratio (Figure 1(d)) was also a potential predictor for ARDS development (cutoff value: 143.9, AUC: 0.618, 95% CI: 0.518–0.718, sensitivity: 53.7%, specificity: 67.6%, and *P*=0.033).

As mentioned above, potential risk factors including age, SOFA score and LIS, body temperature, heart rate, SBP, SpO_2_, BUN, creatinine, lactate, endocan, CRP, and PaO_2_/FiO_2_ were all enrolled in univariate logistic regression analysis. Those factors with a *P* value < 0.1 by the univariate logistic analysis were verified by the multivariate logistic analysis. As shown in Table 3, our final results suggested plasma endocan expression as the only independent risk factor for ARDS development in SP patients (OR: 1.57, 95% CI: 1.14–2.25, *P*=0.021).

## 4. Discussion

SP, a major cause for ICU admission, is closely associated with increased morbidity and mortality even with appropriate support and antibiotic therapy [16]. ARDS is a severe lung injury with acute respiratory failure, and insufficient effective therapy will result in high mortality [4]. SP has been widely recognized as the predominant cause of ARDS, and ARDS often develops due to the failure of therapy for SP [17]. Thus, the early prediction for ARDS in SP patients can probably aid in the prevention and intervention from fatal ARDS development. This present study indicated plasma endocan expression as a potential predictor for ARDS development during the hospital stay in SP patients. The significant association between endothelial glycocalyx biomarkers (endocan, syndecan-1, and hyaluronan) and development of respiratory failure has also be explored by Smart et al. [18], who observed in a smaller series of pneumonia-induced septic patients. However, their results suggest syndecan-1 as a strong predictor of respiratory failure instead of endocan, which is not in line with our results. In our opinion, the variable timing of blood sampling, the therapeutic intervention and illness status can explain the different conclusions.

A previous study conducted in in vitro endothelial cells has reported an increased endocan secretion by the stimulation of tumor necrosis factor-alpha and lipopolysaccharide [14]. Higher circulating endocan levels are also observed in the septic patient in comparison with those with systemic inflammatory response syndrome (SIRS) [14]. Furthermore, a close association between endocan concentrations and severity and mortality is revealed by a study of 60 septic patients [19]. As for those patients undergoing cardiac surgery, endocan instead of procalcitonin or CRP has been suggested as a useful early marker for postoperative pneumonia [20]. Taken together, our observations as well as these results suggest that endocan correlates with the overall severity of acute systemic inflammation.

Lower endocan concentrations on admission are observed in trauma patients who develop ARDS than those without ARDS onset [21]. Another study has revealed a close association between lower endocan expressions on admission and respiratory failure development in septic patients [22]. This association was also confirmed in another study by Gaudet et al. [23], performed in an independent and larger cohort of septic patients. Some colleagues hold the view that endocan measurement is relatively stable and accurate in ICU patients, and aberrant endocan expressions are observed in ICU patients with ARDS development [24]. The results mentioned above suggest that insufficient endocan levels observed in such cases prior to the development of lung injury seem to predict a higher risk of respiratory failure.

As illustrated by our results, the increased plasma endocan expression could potentially serve as a predictor for ARDS development, which enriched the limited literature in this area. As we know, the literature about endocan expressions in SP or ARDS is relatively limited. Increased circulating endocan levels have been proved to be closely associated with progression into ARDS during the follow-up of septic patients [25], which is in support of our conclusions. A recent study has reported that increased circulating endocan concentrations over sepsis follow-up closely correlate with the progression of organ dysfunction and ARDS development [25], which was quite in accordance with our conclusions. A previous study conducted in mainly sepsis-induced ARDS patients has revealed that elevated endocan concentrations significantly correlate with prolonged mechanical ventilation, more severity, and higher mortality rate [10]. In our opinion, the difference in timing of the endocan level may probably explain the apparent contradiction between studies showing high levels of endocan in ARDS patients and some others showing a low level of endocan in septic patients, predictive of ARDS occurrence. In summary, our results, as well as some other reports, suggest that increased endocan levels in constituted lung inflammation (pneumonia and ARDS) seem to correlate with poor outcomes.

## 5. Conclusions

Our results identified endocan as an independent predictor of ARDS in SP patients. Based on the results, we suggest that endocan value might be used to improve the efficiency of existing predictive scores of ARDS or that it could be combined with other biomarkers of ARDS.

## Figures and Tables

**Table 1:** Demographic and clinical characteristics of SP patients with or without ARDS development.

Parameters	SP with ARDS development		*P* value
	Yes (*n* = 37)	No (*n* = 108)	
Age (years)	61.3 ± 10.4	56.4 ± 9.8	0.011
Gender (*n*, %)			0.12
Male	23 (62.2%)	51 (47.2%)	
Female	14 (37.8%)	57 (52.8%)	
Current smokers (*n*, %)	12 (32.4%)	23 (21.3%)	0.17
Clinical scores			
APACHE II	16.7 ± 5.1	16.5 ± 4.7	0.83
SOFA	4.4 ± 1.9	5.2 ± 2.1	0.043
LIS	2.5 ± 0.7	2.2 ± 0.6	0.013
Comorbidities			
Diabetes	7 (18.9%)	27 (25.0%)	0.45
Metabolic diseases	2 (5.4%)	8 (7.4%)	0.68
Renal diseases	7 (18.9%)	21 (19.4%)	0.94
Cardiovascular diseases	6 (16.2%)	17 (15.7%)	0.95
Sepsis (%)	19 (51.4%)	43 (39.8%)	0.22
Body temperature (°C)	37.5 ± 0.9	37.1 ± 0.8	0.012
Respiratory rate (bpm)	24.3 ± 7.8	23.8 ± 8.4	0.75
Heart rate (bpm)	112.4 ± 19.9	103.4 ± 17.8	0.011
SBP (mmHg)	110.1 ± 20.1	119.8 ± 24.3	0.031
DBP (mmHg)	68.4 ± 15.9	70.4 ± 16.1	0.51
SpO_2_ (%)	94.1 ± 5.5	96.2 ± 4.8	0.029
Pleural effusion (%)	23 (62.2%)	78 (72.2%)	0.25
Treatment strategies during the overall ICU stay (%)			
Corticosteroids	21 (56.8%)	75 (69.4%)	0.16
Blood product transfusion	6 (16.2%)	11 (10.2%)	0.33
MV	36 (97.3%)	100 (92.6%)	0.31

SP, severe pneumonia; ARDS, acute respiratory distress syndrome; APACH, acute physiology and chronic health evaluation; SOFA, sequential organ failure assessment; LIS, lung injury score; SBP, systolic blood pressure; DBP, diastolic blood pressure; SpO_2_, oxygen saturation of pulse oximetry; MV, mechanical ventilation. *P* values were calculated by Student's *t*-test, Mann–Whitney *U*-test, chi-squared test, or Fisher's exact test. ^*∗*^*P* < 0.05.

**Table 2:** Laboratory tests of SP patients with or without ARDS development.

Laboratory tests	SP with ARDS development		*P* value
	Yes (*n* = 37)	No (*n* = 108)	
Hemoglobin (g/L)	103.1 ± 25.1	106.5 ± 30.1	0.54
Hematocrit (%)	33.4 ± 6.4	32.7 ± 5.8	0.54
Platelet (109/L)	171.7 ± 102.2	180.4 ± 110.8	0.68
Albumin (g/L)	29.1 ± 6.3	27.4 ± 7.5	0.22
ALT (IU/L)	65.5 ± 88.1	45.4 ± 66.8	0.15
AST (IU/L)	58.4 ± 102.3	38.4 ± 77.4	0.22
BUN (mmol/L)	11.5 ± 6.2	9.3 ± 5.5	0.044
Creatinine (µmol/L)	101.2 ± 48.5	78.4 ± 50.1	0.017
Lactate (mmol/L)	2.2 ± 1.5	1.5 ± 1.1	0.003
Endocan (ng/mL)	13.4 ± 7.2	8.5 ± 4.1	<0.001
T cell immunity (%)			
CD3+	59.4 ± 14.2	57.1 ± 16.3	0.45
CD4+	27.1 ± 12.4	27.9 ± 14.5	0.76
CD8+	28.9 ± 11.7	25.4 ± 12.2	0.13
Inflammatory biomarkers			
CRP (ng/L)	129.5 ± 152.4	85.7 ± 116.7	0.034
Interleukin-6 (ng/mL)	1035.4 ± 783.2	886.5 ± 568.7	0.22
Procalcitonin (ng/mL)	3.5 ± 5.3	4.7 ± 7.1	0.35
Arterial blood gas			
pH	7.40 ± 0.09	7.38 ± 0.08	0.21
PaCO_2_ (mmHg)	40.1 ± 13.1	42.2 ± 12.8	0.396
HCO_3_^−^ (mmol/L)	23.6 ± 5.1	24.1 ± 5.3	0.62
PaO_2_/FiO_2_ (mmHg)	129.3 ± 35.8	152.1 ± 45.1	0.006

SP, severe pneumonia; ARDS, acute respiratory distress syndrome; ALT, alanine aminotransferase; AST, aspartate aminotransferase; BUN, blood urea nitrogen; CRP, C-reactive protein; PaCO_2_, partial pressure of arterial carbon dioxide; HCO_3_^−^, bicarbonate ion; PaO_2_/FiO_2_, ratio of partial pressure of arterial oxygen to fraction of inspired oxygen. *P* values were calculated by Student's *t*-test or Mann–Whitney *U*-test. ^*∗*^*P* < 0.05.

**Table 3:** Risk factors for ARDS development by univariate and multiple logistic regression analysis in SP patients.

Parameters	Univariate/multivariate			*P* value
	OR (95% CI)	*P* value	OR (95% CI)	
Age	1.04 (0.83–1.36)	0.61		
SOFA score	0.85 (0.62–1.25)	0.42		
LIS	1.24 (0.91–1.78)	0.17		
Body temperature	1.12 (0.78–1.59)	0.52		
Heart rate	1.47 (1.03–2.13)	0.021	0.99 (0.73–1.41)	0.75
SBP	1.22 (0.85–1.79)	0.25		
SpO_2_	1.03 (0.81–1.38)	0.77		
BUN	1.17 (0.73–1.65)	0.61		
Creatinine	1.04 (0.73–1.45)	0.082	1.17 (0.72–1.88)	0.65
Lactate	0.73 (0.53–0.99)	0.018	1.03 (0.66–1.57)	0.51
Endocan	1.65 (1.18–2.42)	0.012	1.57 (1.14–2.25)	0.021
CRP	1.21 (0.98–1.57)	0.067	1.22 (0.95–1.68)	0.17
PaO_2_/FiO_2_	1.86 (1.17–3.12)	0.024	1.57 (0.79–2.81)	0.14

SP, severe pneumonia; ARDS, acute respiratory distress syndrome; SOFA, sequential organ failure assessment; LIS, lung injury score; SBP, systolic blood pressure; SpO_2_, oxygen saturation of pulse oximetry; BUN, blood urea nitrogen; CRP, C-reactive protein; PaO_2_/FiO_2_, ratio of partial pressure of arterial oxygen to fraction of inspired oxygen; CI, confidence interval; OR, odds ratio. ^*∗*^*P* < 0.05.

## Data Availability

The data used to support the findings of this study are available from the corresponding author upon request.
